# C-reactive protein and pentraxin-3 binding of factor H-like protein 1 differs from complement factor H: implications for retinal inflammation

**DOI:** 10.1038/s41598-017-18395-7

**Published:** 2018-01-26

**Authors:** Maurice Swinkels, Justine H. Zhang, Viranga Tilakaratna, Graeme Black, Rahat Perveen, Selina McHarg, Antonio Inforzato, Anthony J. Day, Simon J. Clark

**Affiliations:** 1 Wellcome Trust Centre for Cell-Matrix Research, Division of Cell-Matrix Biology and Regenerative Medicine, School of Biological Sciences, Faculty of Biology, Medicine and Health, University of Manchester, Manchester Academic Health Sciences Centre, Oxford Road, Manchester, M13 9PT UK; 2 Manchester Royal Eye Hospital, Central Manchester University Hospitals NHS Foundation Trust, Manchester Academic Health Science Centre, Manchester, M13 9WL UK; 3 Division of Evolution and Genomic Science, School of Biological Sciences, Faculty of Biology, Medicine and Health, University of Manchester, Oxford Road, Manchester, M13 9PT UK; 4 Manchester Centre for Genomic Medicine, Saint Mary’s Hospital, Central Manchester University Hospitals NHS Foundation Trust, Manchester, UK; 5 Humanitas Clinical and Research Center, Via Manzoni 56, Rozzano, 20089 Italy; 6 Department of Medical Biotechnologies and Translational Medicine, University of Milan, Via Vanvitelli 32, Milan, 20129 Italy; 7 Present Address: Department of Hematology, Erasmus University Medical Centre, ‘s-Gravendijkwal 230, 3015 CE Rotterdam, The Netherlands

## Abstract

Retinal inflammation plays a key role in the progression of age-related macular degeneration (AMD), a condition that leads to loss of central vision. The deposition of the acute phase pentraxin C-reactive protein (CRP) in the macula activates the complement system, thereby contributing to dysregulated inflammation. The complement protein factor H (FH) can bind CRP and down-regulate an inflammatory response. However, it is not known whether a truncated form of FH, called factor H-like protein 1 (FHL-1), which plays a significant regulatory role in the eye, also interacts with CRP. Here, we compare the binding properties of FHL-1 and FH to both CRP and the related protein pentraxin-3 (PTX3). We find that, unlike FH, FHL-1 can bind pro-inflammatory monomeric CRP (mCRP) as well as the circulating pentameric form. Furthermore, the four-amino acid C-terminal tail of FHL-1 (not present in FH) plays a role in mediating its binding to mCRP. PTX3 was found to be present in the macula of donor eyes and the AMD-associated Y402H polymorphism altered the binding of FHL-1 to PTX3. Our findings reveal that the binding characteristics of FHL-1 differ from those of FH, likely underpinning independent immune regulatory functions in the context of the human retina.

## Introduction

**Figure 1:**
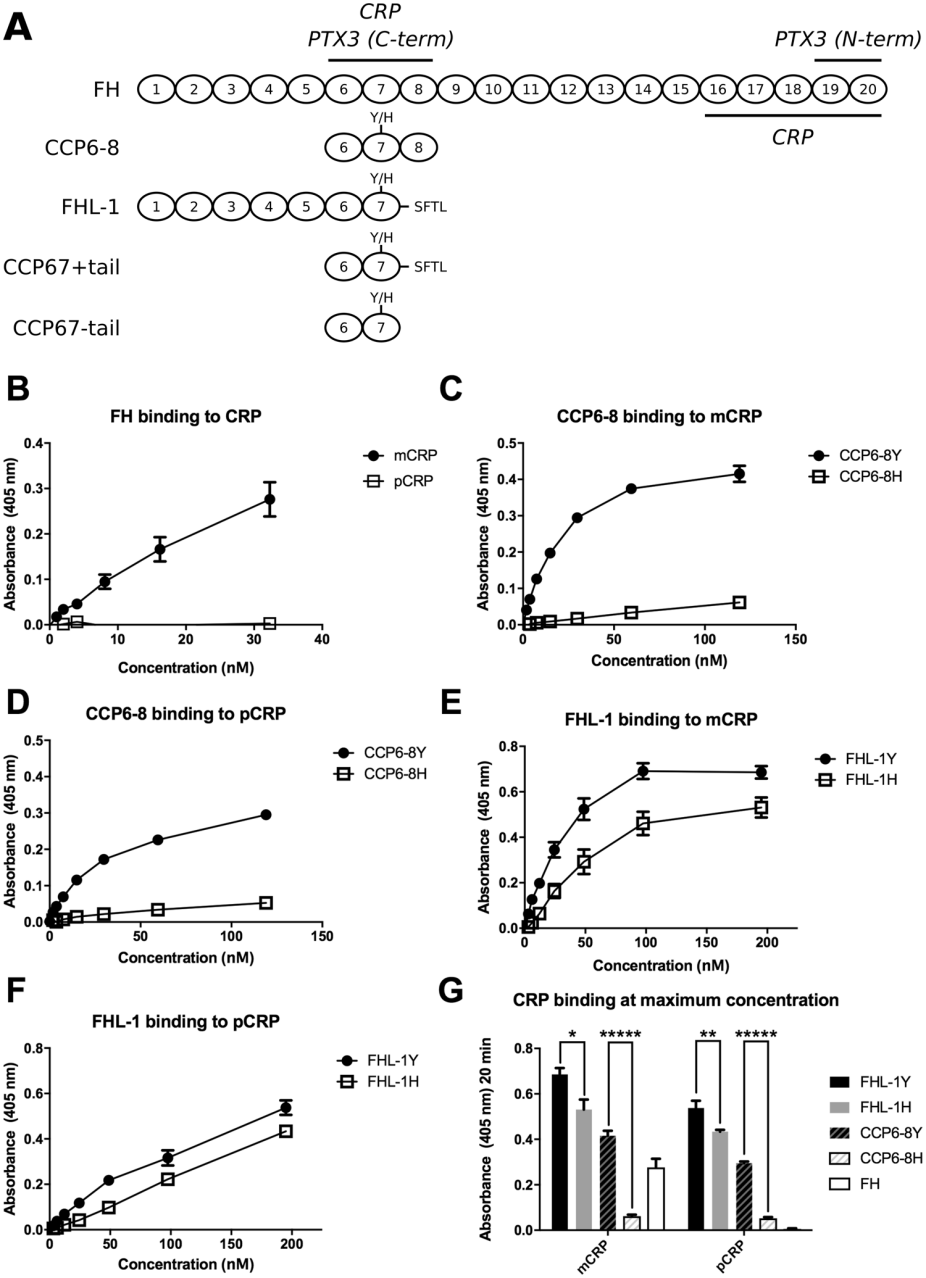
FHL-1, but not FH, binds to both monomeric and pentameric CRP. (**A**) Schematic representations of the FH proteins used in this study and the positions of CRP and PTX3 binding sites on FH/FHL-1; PTX3 binds through both its N- and C-terminal domains^1,28^. (**B**–**F**) A series of FH-based proteins were investigated for binding to immobilized monomeric CRP (mCRP) or pentameric CRP (pCRP) in plate assays: (**B**) Full-length FH from human plasma; (**C**,**D**), binding of the CCP6-8 402Y and 402 H variants to mCRP (**C**) and pCRP (**D**); (**E**,**F**), binding of the FHL-1 402Y and 402 variants to mCRP(**E**) and pCRP (**F**). (**G**) The maximum binding values for CCP6-8, FHL-1, and FH are re-plotted from the experiments in (**B**–**F**) to highlight the significant effect of the Y402H polymorphism; here paired Student’s t tests were run on selected data as shown (*p < 0.05; **p < 0.01, *****p < 0.00001). All data (mean ± SEM) are combined from two identical experiments performed in quadruplicate (n = 8).

Complement factor H (FH) is an ~155 kDa serum glycoprotein that is mainly synthesized in the liver and is one of the central regulators of the complement system, a protein cascade that plays a crucial role in host innate immunity^1^. Activation of complement, *via* classical, lectin or alternative pathways, leads to a local inflammatory response, generation of membrane attack complexes (MAC) that cause cell lysis, and labels unwanted cells and debris for uptake and disposal by phagocytes; irrespective of how complement is activated, it is the alternative pathway that amplifies the response. As such, the activation and amplification of complement is tightly regulated by a series of cell surface complement regulators including decay accelerating factor (DAF, or CD55), membrane bound co-factor protein (MCP, or CD46), and MAC-inhibitory protein (MAC-IP, or CD59). However, in the case of extracellular matrix structures, including basement membranes, none of these regulators is present and these rely on the recruitment of soluble complement regulators, such as FH, from the blood. Comprising twenty complement control protein (CCP) domains (see Fig. 1A), FH anchors to surfaces primarily through its CCP6-8 and CCP19-20 regions^2^, where it can act as a negative regulator of the alternative pathway; *e.g*. by being a co-factor for complement factor I (FI) that cleaves deposited C3b into inactive C3b (iC3b) thereby down regulating the complement response^1^. A 49 kDa truncated version of FH exists in plasma, termed factor H-like protein 1 (FHL-1), which arises from alternative splicing of the FH gene (*CFH*), and is identical to FH for the first seven CCPs before terminating in a unique four amino acid tail (Fig. 1A)^3,4^. It should be noted that smaller sizes are often quoted for FHL-1, but these are likely to represent the truncated N-terminal CCP1-5 tryptic fragment of FH (37 kDa)^3,4^. Furthermore, CCP containing proteins all run at lower apparent molecular weights when run on reduced gels^5^. Despite lacking the CCP19-20 domains and being present in plasma at lower concentrations than the full-length FH protein (30–50 *vs* 200–800 μg/ml)^6^, FHL-1 is able to regulate complement on surfaces to which it binds *via* its CCP67 domain, depending on the presence of suitable anchoring ligands^7^.

FH and FHL-1 anchor to surfaces through interactions with charged molecules such as the glycosaminoglycan (GAG) chains of proteoglycans and, in the case of FH, also sialic acids^7–9^. The GAGs heparan sulfate (HS) and dermatan sulfate (DS) play a significant role in FH binding to cell surfaces and to extracellular matrices such as the glomeruli basement membrane in the human kidney and Bruch’s membrane in the human eye^2,10^. Indeed, the specificity of FH for patterns of sulfated GAGs is such that the CCP6-8 and 19–20 regions can differentially regulate the binding to host tissues, *i.e*. depending on the population/composition of GAGs expressed therein^2,8,11–13^. This is highlighted by mutations in the CCP6-8 or CCP19-20 regions of FH impairing complement regulation in a tissue-specific manner. For example, a common polymorphism in *CFH*, (Y402H) that leads to a histidine replacing a tyrosine residue in CCP7 of both FH and FHL-1 (at residue 384 or 402 in the mature and pre-protein, respectively)^3,14^, affects the capacity of this anchoring domain to bind HS and DS in Bruch’s membrane in the human eye^2,10,15^; however, it does not affect binding to the glomeruli basement membrane in the kidney, since the latter is mediated by the CCP19-20 region. Furthermore, given that FHL-1 contains only a single GAG-interaction site (in CCPs6-7), and that this protein is the major factor H species present on the human Bruch’s membrane^7^, the Y402H polymorphism has the potential to have a significant impact on FHL-1 binding and thus regulation of complement activation. Indeed, the Y402H polymorphism is strongly associated with an increased risk of age-related macular degeneration (AMD)^16,17^, the leading cause of blindness in the developed world. A hallmark feature of AMD is complement dysregulation in the proximity of Bruch’s membrane that forms part of the outer blood-retinal barrier along with the retinal pigment epithelium (RPE), separating the neurosensory retina from the blood supply in the choroid.

AMD is characterized by complement driven inflammation in the central retina (macula) leading to accumulation of particulate material (termed drusen), cellular damage and the associated loss of central vision^16^. A number of markers of inflammation and complement activation are found deposited in and around Bruch’s membrane during the early stages of disease, including the acute phase, soluble pattern recognition molecule (sPRM), C-reactive protein (CRP)^18^. CRP is a short pentraxin that can activate complement *via* the classical and lectin pathways, thereby promoting opsonization and phagocytosis of apoptotic and necrotic cells^1^. It circulates in blood as a non-covalent, non-inflammatory, pentamer (pCRP), which however dissociates into a pro-inflammatory monomer (mCRP) upon binding to certain surfaces^19^. Dissociation of the pentamer may also occur under particular pathogenic conditions, including absence of Ca^2+^, low pH and high urea concentrations (reviewed in^19^). FH inhibits CRP-mediated complement activation through its binding to the monomeric form of CRP *via* CCPs6-8 and CCPs16-20^20–24^, but does not interact with pCRP^25^. Individuals homozygous for the Y402H polymorphism in *CFH* have been found to have higher levels of CRP in the choroid at the back of the eye^26^, and interestingly the AMD-associated 402H form of FH has been shown to have reduced binding to mCRP^20^ and is less able to inhibit the inflammatory effects of mCRP in RPE cells^27^. Another inflammation-associated pentraxin that has been linked with complement activation is PTX3, where FH is known to bind this long pentraxin *via* its CCP6-8 (via CCP7) and CCP19-20 regions^1,28^; PTX3 enhances the interaction of FH with apoptotic cells leading to their increased opsonization with iC3b^28^. However, unlike CRP^27^, neither the plasma levels of PTX3 nor its gene expression in the RPE/choroid correlate with AMD status^29^. In addition, the Y402H polymorphism does not affect FH binding to PTX3^28^. Interestingly, however, PTX3 expression increases in cultured RPE stimulated with inflammatory cytokines^29,30^ or peroxidated lipids (*i.e*., 4-hydroxynonenal, 4-HNE), and genetic deficiency of PTX3 magnifies complement activation in an animal model of AMD, with increased C3a and IL-1β levels in the RPE, and enhanced accumulation of macrophages in the choroid^31^.

While the interactions of FH with CRP have been well studied, and interactions between FH and PTX3 are starting to be elucidated, very little is known about the binding characteristics of FHL-1 for either pentraxin. Furthermore, the contribution to ligand binding made by the unique C-terminal tail of FHL-1 is poorly understood. Given the likelihood that FHL-1 plays a more significant role in complement regulation than previously thought, particularly in the human eye^5^, we have compared the binding of FH and FHL-1 to both CRP and PTX3, investigated the effect of the AMD-associated Y402H polymorphism on these interactions, and investigated whether the FHL-1 tail contributes to binding.

## Results

### FHL-1 binding to CRP

To investigate the binding properties of FHL-1 for CRP we first validated our solid phase binding assays by using full length FH in the fluid phase and either mCRP or pCRP immobilized onto the surface. Here we found that FH bound mCRP, but did not bind pCRP (Fig. 1B) in agreement with previous studies^25,27^. In order to assess the effect of the AMD-associated Y402H polymorphism on FH binding to CRP we utilized our CCP6-8 recombinant proteins so that we could study this polymorphic region in isolation (*i.e*. in the absence of the secondary binding site for CRP at the C-terminal end of FH)^21^. We found that there was significantly less binding of the AMD-associated 402H variant than the 402Y form of CCP6-8 to immobilized mCRP (Fig. 1C,G), which is consistent with previous studies^20^. However, these truncated recombinant proteins also bound to pCRP (Fig. 1D,G), a phenomenon not shared with the full-length protein (Fig. 1B,G), suggesting interactions with the exposed N- or C- terminal regions of CCPs 6 and 8, respectively. Given that FHL-1 also has a largely exposed C-terminal face on CCP7 (see Fig. 1A), it seemed possible that FHL-1 might also recognize both forms of CRP. Indeed, when we analyzed the 402Y and 402 H variants of FHL-1 a clear binding was observed to both mCRP and pCRP (Fig. 1E,F), where the 402 H form of FHL-1 bound significantly less well to monomeric and pentameric CRP (Fig. 1G). SDS-PAGE verified that the differences in binding of the 402H and 402Y variants were not due to differences in the concentrations (or purity) of the protein preparations used (see Figure S1). Interestingly, FHL-1 (402Y) is a relatively poor competitor against FH binding to mCRP, presumably due to FHL-1 having only one mCRP binding site, while FH has two (see Figure S2).

### Investigating the role of the FHL-1 C-terminal tail on CRP binding

**Figure 2:**
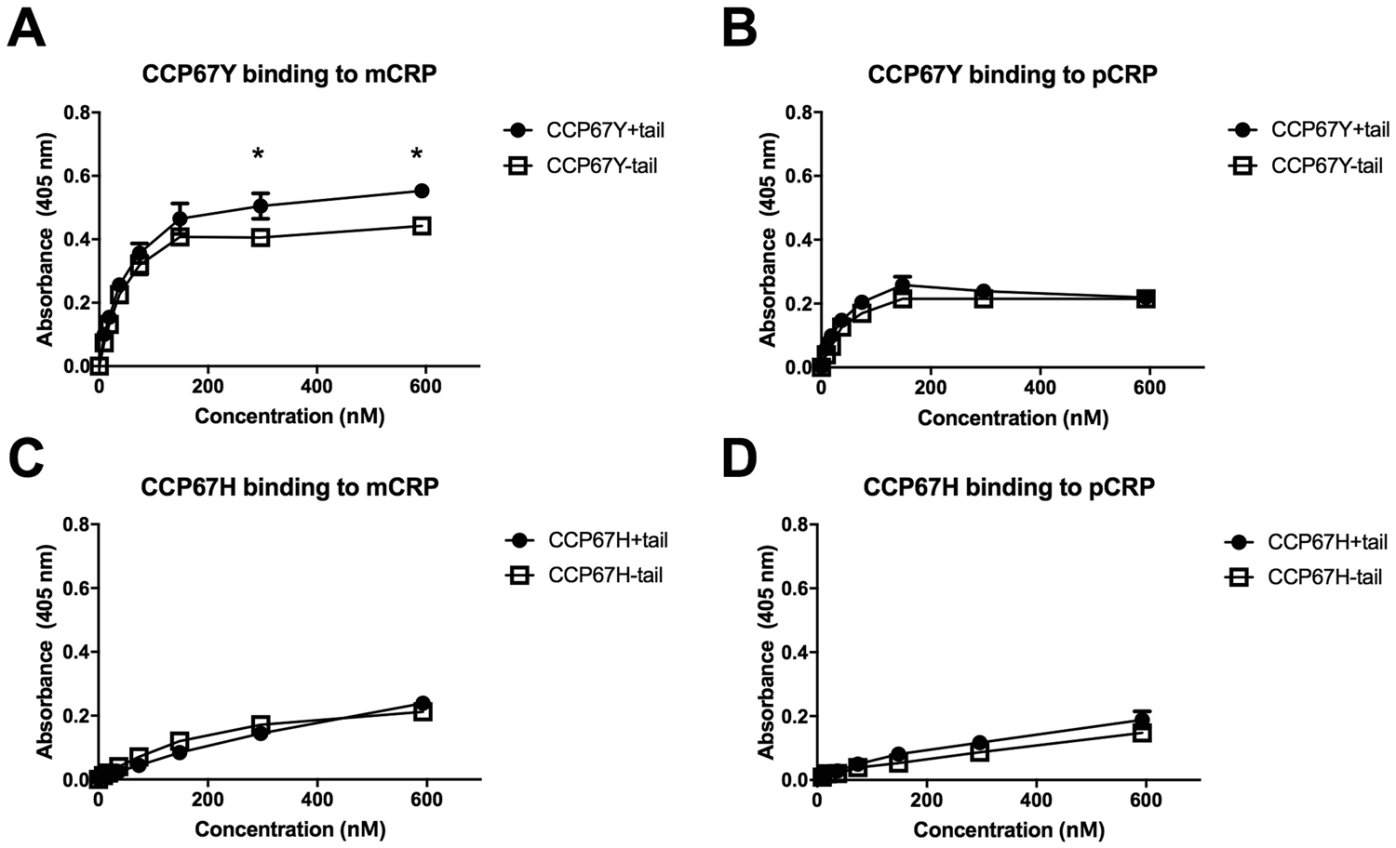
Contribution of the FHL-1 C-terminal tail to CRP binding. Recombinant CCP67 proteins (*i.e*. 402Y and 402 H variants with and without the 4-amino acid C-terminal tail of FHL-1) were used at a range of concentrations to determine their binding to immobilized monomeric CRP (mCRP) or pentameric CRP (pCRP) in plate assays. (**A**) Binding of the 402Y isoform of CCP67 constructs with/without tail to immobilized mCRP. Differences in binding at the two highest concentrations are statistically significant (Student T test, * = p < 0.05). (**B**) Binding of the 402Y isoform of CCP67 constructs with/without a tail to immobilized pCRP. (**C**) Binding of the 402 H isoform of CCP67 constructs with/without a tail to immobilized mCRP. (**D**) Binding of the 402 H isoform of CCP6-7 constructs with/without a tail to immobilized pCRP. Data (mean ± SEM) are combined from two identical experiments performed in quadruplicate (n = 8).

After establishing that FHL-1 bound to both immobilized mCRP and pCRP we then investigated whether the protein’s unique C-terminal tail conferred any effect on these interactions; for this purpose 402 H and 402Y forms of recombinant CCP67 proteins with or without the four amino acid tail were used, where these preparations were validated by SDS-PAGE (Figure S3). Here we found that the tail had a positive influence on the binding of the 402Y form of CCP67 to mCRP (Fig. 2A), where the absence of the tail lead to a statistically significant reduction in the levels of detectable binding at 5 μg/ml and 10 μg/ml concentrations. This effect was not observed when the CCP67 402Y protein was tested with pCRP (Fig. 2B). No effect of the tail on the binding of the 402 H form of CCP67 to either mCRP or pCRP was observed (Fig. 2C,D).

### FHL-1 binding to PTX3 and the role of its C-terminal tail

**Figure 3:**
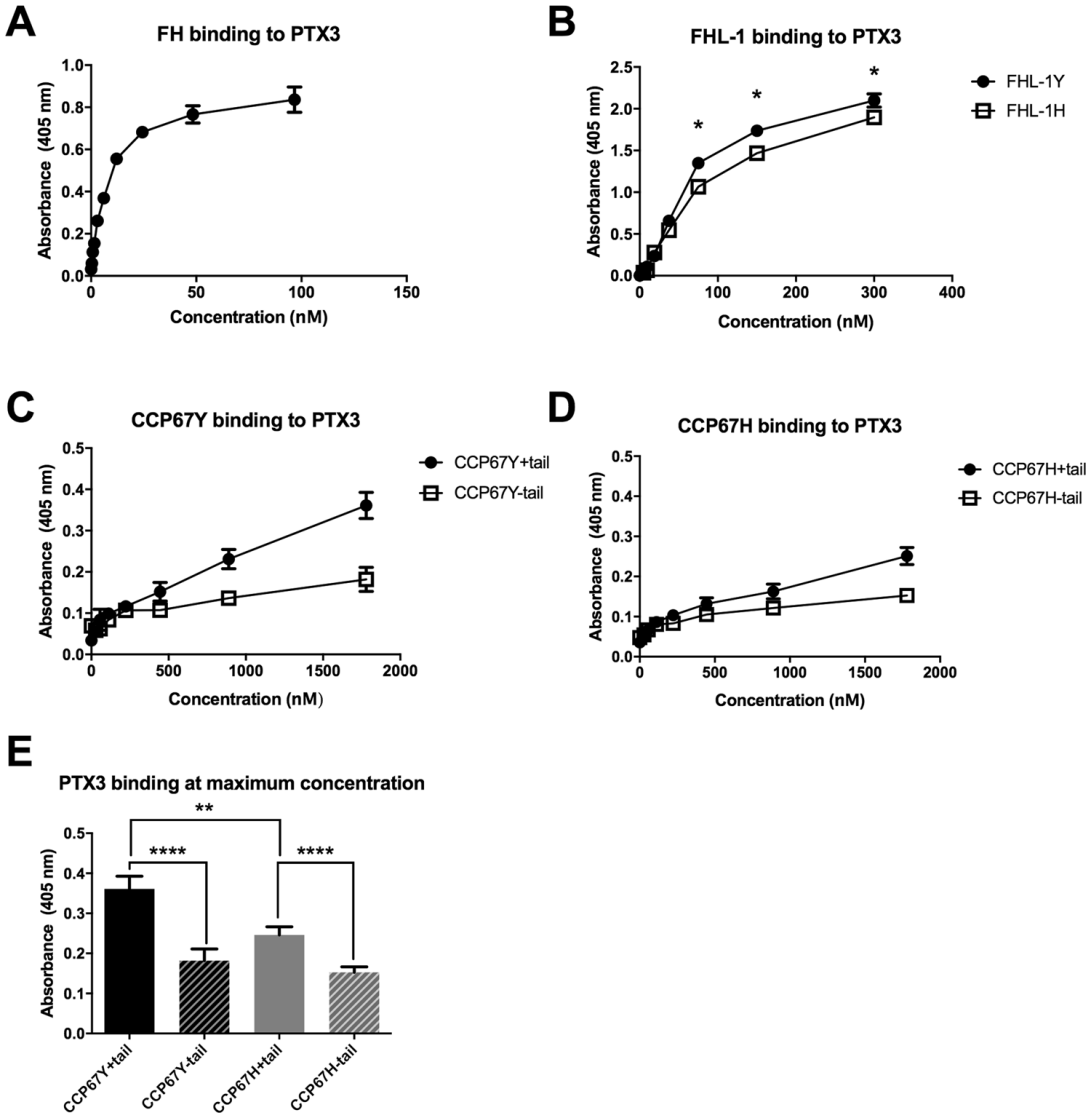
The C-terminal tail of FHL-1 contributes to PTX3 binding. In plate assays, PTX3 was used in the fluid phase to determine its binding to immobilized FH/FHL-1 proteins: (**A**) FH; (**B**) FHL-1: (**C**) CCP67 402Y ( ± tail); and (**D**), CCP67 402 H ( ± tail). (**E**) The maximum binding values obtained from the experiments in (**A**–**D**) were re-plotted to highlight the significant effects between 402Y and 402 H forms of CCP67 ± tail; here statistical analysis (Student’s T test) compared pairs of data as shown (**p < 0.01, ****p < 0.0001). All data (mean ± SEM) are combined from two identical experiments performed in quadruplicate (n = 8) for FH and FHL-1, and three identical experiments performed in quadruplicate (n = 12) for CCP67 constructs. The binding curves in **B** are statistically different when analysed by two-way ANOVA, p = 0.0025, and the binding at the top three individual concentrations are statistically different where *p < 0.001 (Student’s T test).

Given that PTX3 is known to interact with FH, in part *via* a binding site involving CCP7^28^, we investigated whether, like CRP, it also binds to FHL-1. Here, we validated our solid phase binding assay by showing that the full-length FH interacted with immobilized PTX3 (Fig. 3A), as was expected, and under the same conditions determined that FHL-1 also interacts with PTX3 (Fig. 3B). The binding of FHL-1 to PTX3 appears to be of similar affinity to that of FHL-1 binding mCRP as evidenced in competition assays where ~50% inhibition of FHL-1 binding to immobilized mCRP is achieved with equimolar amounts of PTX3 (see Figure S4). Comparison of the 402H and 402Y variants of FHL-1 showed that the Y402H polymorphism causes a small but significant reduction in binding (*p* = 0.0025; by two-way ANOVA). The C-terminal tail of FHL-1 was found to make a significant contribution to the interaction with PTX3 in the context of both the 402Y and 402H variants (Fig. 3C–E).

### Localization of CRP and PTX3 in non-AMD and AMD donor eyes

**Figure 4:**
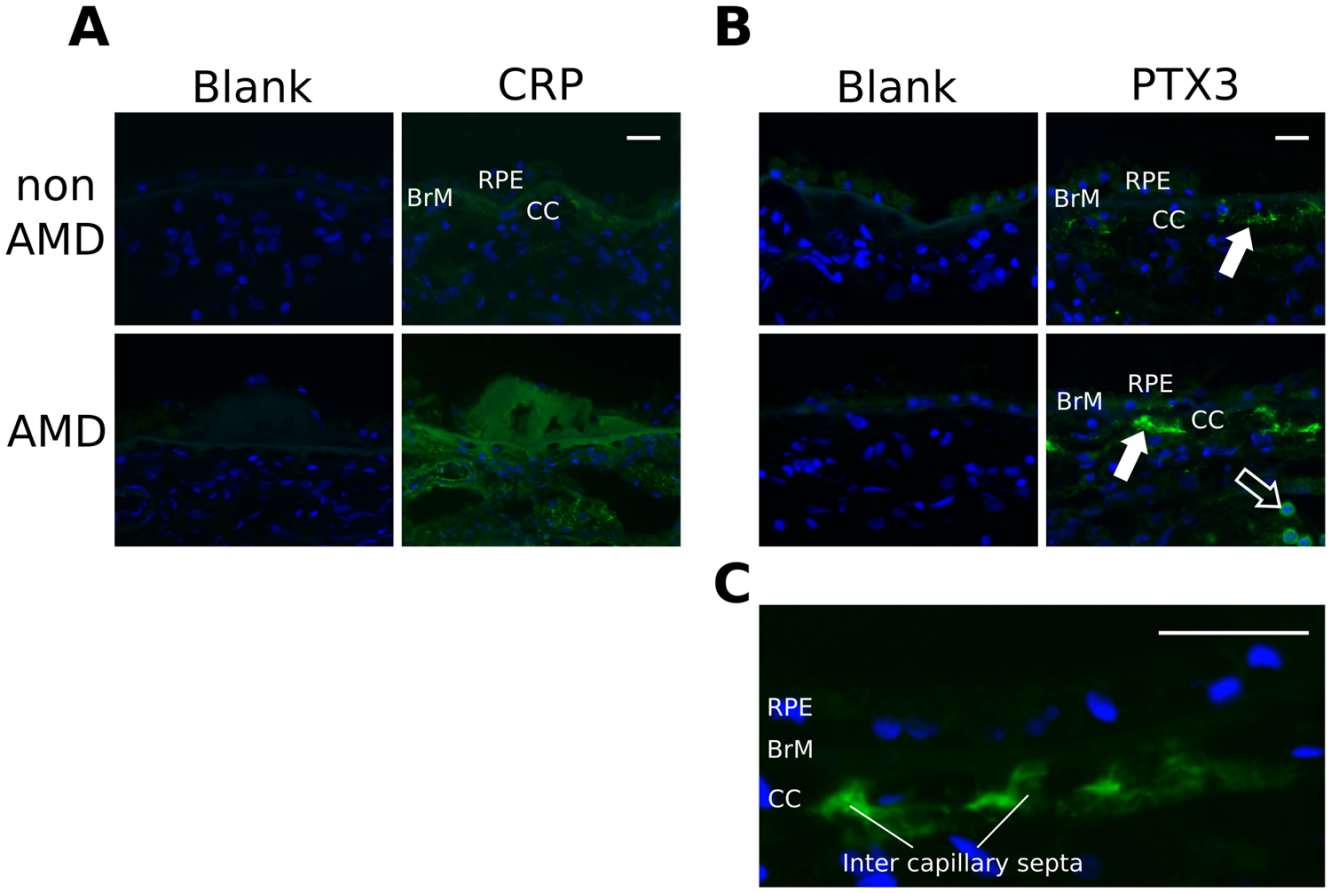
Immunofluorescent staining of CRP and PTX3 in the macula of human donor eyes. Representative images of five (for CRP staining) and eighteen (PTX3) donor tissues are shown. (**A**) CRP staining in the macula region of non-AMD and AMD eyes (green) can be seen in the Bruch’s membrane (BrM) and choriocapillaris (CC), which underlie the retinal pigment epithelium (RPE); CRP staining was used as a control, given that it is known to accumulate in these locations during AMD. (**B**) Staining for PTX3 (green) can be seen in the stroma of the choriocapillaris (solid white arrows) and, in the case of AMD donors, cells within the blood vessels of the choroid also stain positive for PTX3 (open white arrow). (**C**) A higher magnification image of PTX3 staining (green) in the intercapillary septa of the choriocapillaris. In all cases blue nuclear staining is achieved with DAPI, and scale bars represent 10μm.

**Figure 5:**
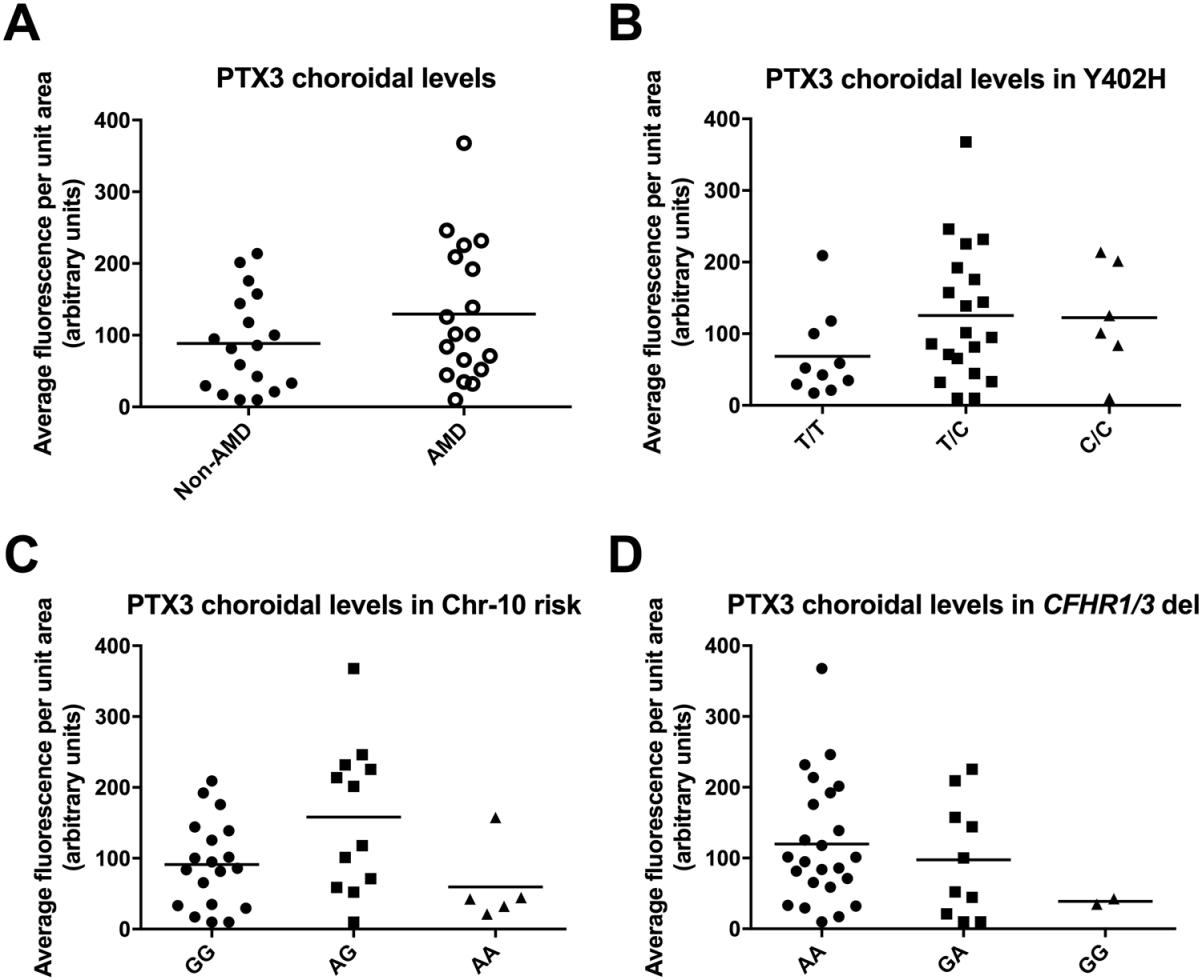
Analysis of PTX3 staining in the choroid of human donor eyes stratified for AMD and AMD-risk genotypes. The average fluorescence intensity for PTX3 staining in the choroid of eighteen non-AMD and eighteen AMD donor tissues is shown. (**A**) PTX3 staining in the choroid of AMD eyes showed a trend towards increased levels when compared to age-matched control eyes, but did not reach statistical significance. (**B**) All donors (n = 36) were stratified based on the Y402H AMD-risk genotype and a trend was seen for increasing PTX3 staining when going from T/T (non-risk; 402Y/402Y homozygotes) to T/C to C/C (high risk; 402 H/402H homozygotes) genotypes. (**C**) PTX3 staining levels did not appear to correlate with chromosome 10 (Chr-10) non-risk (G/G) or AMD risk (A/T, A/A) genotypes. (**D**) The *CFHR1/3* gene deletion (*CFHR1/3* del) showed a trend towards a reduction in PTX3 staining with AMD-non-risk (GA, GG) genotypes, but did not to reach significance. Statistical analysis was performed using one-way ANOVA.

Since FHL-1 is able to interact with CRP and PTX3, and this truncated form of FH has been implicated as the predominant soluble complement regulator in Bruch’s membrane^7^, we stained for both of these related pentraxins in human eye tissues from donors with and without AMD. Consistent with previous studies^18,26^, CRP levels were found to be greater in AMD compared to non-AMD donor tissues, with staining associated with Bruch’s membrane and the underlying fenestrated blood vessels of the choriocapillaris (Fig. 4A). Staining for PTX3, which to our knowledge has not been thoroughly investigated in human donor eyes, revealed patches of positive immunoreactivity underneath Bruch’s membrane, in the intercapillary septa of the choriocapillaris and in the choroid (Fig. 4B,C). When the levels of PTX3 staining were quantitated (based on fluorescence intensity measurements), there was a slight trend towards increasing PTX3 levels in the AMD compared to non-AMD samples, however, this was not statistically significant for either the choroid (Fig. 5A) or choriocapillaris (Figure S5A). Furthermore, no statistically significant difference was seen for PTX3 levels when donor tissues were stratified on the basis of the Y402H polymorphism. Given the trend towards increasing PTX3 with AMD we stratified our samples for another high-risk genotype for AMD (SNPs in Chromosome 10 (Chr-10) around the *ARMS2/HTRA1* genes) as well as a common AMD-protective genotype that includes the deletion of the *CFHR1* and *CFHR3* genes (Figs 5B–D and S5B–D). Neither showed a significant effect when analyzed by one-way ANOVA. The presence of a small number of luminal leukocytes that gave a strong fluorescent signal were detected in some of the donors, however this only occurred in five of the 18 non-AMD donors, and four of the AMD cases, thus their presence did not alter the overall comparison of these samples.

## Discussion

In this study we describe for the first time that FHL-1, a truncated form of FH, can bind to CRP and PTX3, prototypes of the short and long pentraxins, respectively. FHL-1 was found to interact with both pro-inflammatory, monomeric, CRP and the circulating pentameric form, as well as the long pentraxin PTX3, which we have shown here to be present in the ECM of the choroid and choriocapillaris of eyes from AMD and non-AMD donors. Importantly, the 402Y variant of FHL-1 was found to bind significantly better to both CRP isoforms compared to the 402H variant, where as described below the altered binding to mCRP may be of particular relevance to AMD.

A unique feature of FHL-1 is the presence (at its C-terminus) of a 4 amino acid sequence on the exposed face of CCP7 that is not present in the full-length protein, or indeed some recombinant mimics of this protein^23,24,28,32–34^. Unlike FH, FHL-1 was found to bind pCRP in addition to mCRP, but the presence or absence of the tail did not have a major effect on binding when analyzed in the context of C-terminal CCP67 constructs that recapitulate the interaction of FHL-1 with CRP (Fig. 2). There was however a small, but statistically significant, reduction in the binding of the 402Y form of CCP67 with mCRP, when the tail was absent, suggesting that this sequence might play some minor role for this variant. Moreover, the tail was not implicated in the binding of the 402H variant of FHL-1 to mCRP or indeed the interaction of either variant of FHL-1 with pCRP, suggesting that other amino acids on the exposed face of CCP7 mediate these interactions. Interestingly, unlike CRP, the C-terminal tail of FHL-1 does appear to play an important role in its binding to PTX3 (Fig. 3C–E), where the absence of the SFTL sequence (in CCP67 constructs) led to a pronounced (and significant) reduction in the observed interaction: something that has not been reported previously. In addition, the AMD-associated Y402H polymorphism appeared to alter the binding of FHL-1 to PTX3 (where the 402Y allotype binds better than 402H), although to a lesser extent than that described for CRP. This observation is inconsistent with a previous report indicating that the interaction of full length FH with PTX3 is not affected by the Y402H polymorphism^28^. However, it is likely that the interaction of FHL-1 with PTX3 is more sensitive to the Y402H polymorphism, because the polymorphism occurs in the only PTX3 binding site present in FHL-1, unlike in FH where a secondary binding site exists in CCPs19-20.

Given the interaction between FHL-1 and PTX3 we investigated the localization of this pentraxin in human donor eye tissues. PTX3 is known to bind FH at CCP7 and CCP19-20^28^, is able to modulate the classical and lectin pathways of complement^35^, and is expressed locally at sites of inflammation^29^, such as in RPE cells^30,36^. The C-terminal domain of PTX3 is homologous to CRP and binds CCP7, while the N-terminal domain binds CCP19-20^28^. PTX3 expression has been documented in a mouse model of AMD, where it co-localizes with FH in RPE and Bruch’s membrane. Furthermore, previous immunohistochemical staining of PTX-3 in the macula of an AMD patient detected this pentraxin in multiple layers of the retina, including Bruch’s membrane and the basement membrane of both RPE and choriocapillaris^37^. Consistent with previous reports, here we show that the PTX3 protein is present in the human macular tissue of AMD donors, however, for the first time, we documented the presence of this pentraxin in the macula of non-AMD donors. In this regard, PTX3 is well established to be expressed in inflammatory and inflammation-like conditions, and constitutive expression of the protein has only been described in cells from human amniotic membrane^38^ and in a mouse lymphatic endothelial cell line^39^. Our observation suggests a physiological role for PTX3 in the human macula, where the protein, locally and constitutively made by the choroid and chorocapillaris endothelium (Fig. 4)^37^, might contribute to tissue homeostasis, *e.g*. by participating in the control of complement activation. In this respect, PTX3 has been recently proposed to act as an anchoring site for FH in Bruch’s membrane and RPE, where it limits complement-dependent inflammatory response in a mouse model of oxidative stress-induced AMD^31^. This mechanism might also operate under physiological (non-AMD) conditions, to preserve integrity and functionality of the retinal tissue. Quantitation of staining revealed a trend towards higher PTX3 levels in AMD eyes and hinted at a possible relationship with AMD-associated SNPs, *i.e*. those responsible for the deletion of factor H related genes 1 and 3 (*CFHR1-3*) and the Y402H coding change (Figs 5 and S5); however, additional analysis with a greater number of donor samples would be required to investigate this further.

Dissociation of the pCRP into the pro-inflammatory mCRP is known to occur upon cell and matrix surfaces^19^, indicating that mCRP may be a tissue bound form while pCRP remains in circulation. Indeed, it has been shown recently that the majority of CRP found in processed human macula tissue sections is monomeric^40^, although undoubtedly non-deposited pCRP will be present within the lumen of blood vessels of the choroid *in vivo*. The exact mechanism of this dissociation is unclear, but may involve phospholipids such as lysophosphatidylcholine^19,41^. Bruch’s membrane acts as a biological filter to allow exchange of nutrients and waste products (but not cells or large macromolecules, *i.e*. above ~100 kDa) between the blood supply in the choroid and the cells in the neurosensory retina and its size exclusion properties are affected by age^42,43^. Therefore, the CRP present in Bruch’s membrane (Fig. 4) is most probably mCRP (~25 kDa), because of the likely lack of permeability to the larger pCRP (~125 kDa). Since mCRP is predominantly generated on cell surfaces^19,41^, it has been suggested that it is transported across Bruch’s membrane by way of cell membrane vesicles; thus, having the potential to become directly associated or trapped within this extracellular matrix. In this context, given that FHL-1 is the predominant form of complement regulator in BrM and intercapillary septa^7,44^ (FHL-1 can diffuse through the membrane while FH can not) and given that mCRP is also present in these locations^18,22,45^ (see Fig. 4A), it seems probable that FHL-1 will bind to mCRP and dampen any subsequent complement activation and the inflammatory response. This is unlike the binding of FHR-1 to mCRP, which has been found to promote complement activation^46^, because FHL-1 retains the capacity to act as a co-factor for the FI mediated cleavage of C3b, whereas FHR-1 does not. Our finding that the Y402H coding change reduces the capacity of FHL-1 to bind to mCRP (Fig. 1E) is important since it could explain how this AMD-associated polymorphism results in impaired complement regulation in or around BrM, leading to local inflammation that contributes to AMD disease progression.

Besides complement activation and inflammation, angiogenesis and vascular remodeling are well known phenomena in AMD pathogenesis, particularly in wet AMD^47^. PTX3 inhibits FGF2-dependent angiogenesis^48–50^ and is also involved in matrix remodeling by crosslinking complexes of the polysaccharide hyaluronan covalently linked with so-called heavy chains, where this occurs during physiological processes (such as ovulation) and during inflammation^51–53^. Heavy chains are transferred onto hyaluronan from the protein inter-α-inhibitor (IαI), which is catalyzed by the inflammation-associated protein TNF-stimulated gene-6 (TSG-6)^52^; TSG-6 may also inhibit the anti-angiogenic properties of PTX3^50^. Hyaluronan is present throughout the macula, *e.g*. being present in the neurosensory retina, RPE, Bruch’s membrane and choroid^54^, however, the presence and localization of IαI and TSG-6 in this area of the eye has not been studied. We also observed PTX3 staining in yet-unidentified cells, which could be leukocytes (Fig. 4). PTX3 is locally produced by various cells of the immune system such as monocytes, macrophages, dendritic cells and neutrophils^36,55^; furthermore, endothelial cells, smooth muscle cells, epithelial cells and fibroblasts also express PTX3^36^. Judging by the size and shape of the colocalizing cells (5-10 µm), these could be neutrophils, macrophages or monocytes. However, additional studies will be necessary to investigate this further.

The emergence of the pentraxin-family members CRP and PTX3 as potential mediators of complement in the eye could explain some of the molecular mechanisms of the disease. Genetic risk factors for AMD, such as the Y402H polymorphism, provide increasing evidence of the involvement of inflammatory processes in AMD in which FH and potentially FHL-1 may play a central role^16,56–58^. The Y402H polymorphism in the *CFH* gene results in an impaired ability to anchor FH/FHL-1 to cell surfaces and ECM *via* heparan sulfate^10^, as well as reduced binding to inflammatory mediators like CRP and, as demonstrated here, PTX3^20,27,59^. Based on the data in this report, we hypothesize that the Y402H polymorphism reduces the ability of FH and FHL-1 to bind inflammatory mCRP and PTX3 (for FHL-1) in choroid (FH) and Bruch’s membrane (FHL-1). As a result, ECM structures like Bruch’s membrane will be insufficiently protected from inflammation and this would contribute to AMD pathogenesis. In addition to inflammatory processes, both CRP and PTX3 affect angiogenesis and vascular remodeling. PTX3 may inhibit angiogenesis in some cases^48,49^, which is affected by the PTX3 binding protein TSG-6^50^. At the same time, mCRP is able to induce CXCL8 and CCL2 expression in cultured RPE cells, cytokines which are both highly involved in choroidal neovascularization in AMD^60,61^. FH has been shown to inhibit this mCRP-induced expression in RPE cells^27^.

Our work indicates that the functional activities of FHL-1 are unlikely to simply replicate those of FH, and these proteins must be considered separately to get a true understanding of how complement activation and homeostasis are regulated.

## Materials and Methods

### Protein reagents and antibodies

For plate assay experiments, commercial preparations of full-length FH and CRP, both purified from pooled human plasma (Sigma-Aldrich, Poole, UK), were used. Recombinant factor H proteins, *i.e*. FHL-1 (402Y and 402H variants), CCP6-8 (402Y/402YH) and CCP67 (402Y/402H ± tail) were made in-house using previously published protocols^7,33^. Human PTX3 was expressed in PerC6 cells and purified as described previously^62^, and was subject to buffer-exchange (10 mM Tris, 150 mM NaCl pH 7.5) by size exclusion chromatography with multiple angle laser light scattering (SEC-MALLS) before use; this verified that the protein used was octameric^48^. In plate assays, the murine OX23 monoclonal antibody (Abcam, Cambridge, United Kingdom) was used to detect FH binding while a rabbit polyclonal antiserum was raised against a peptide from CCP7 of FH/FHL-1 (H-CGYNQNHGRKFV-NH_2_ coupled to Diphtheria Toxoid as the carrier protein) by Mimotopes (Clayton, Australia) and used to detect the recombinant factor H proteins (*i.e*. FHL-1, CCP6-8 and CCP67 ± tail); in control experiments the polyclonal was demonstrated to have identical binding to the 402H and 402Y variants. In plate assays, bound PTX3 was detected using a rabbit polyclonal antibody^63^.

For the fluorescent staining of human donor eye tissue, endogenous CRP was detected with a rabbit anti-CRP polyclonal antibody (cat. No. 235752; Calbiochem, Watford, UK), which does not distinguish mCRP from pCRP, and PTX3 was visualized using the same anti-PTX3 polyclonal antibody as described above. In both cases bound primary antibody was detected with an AlexaFluor488 conjugated goat anti-rabbit antibody (Life Technologies, Paisley, UK).

### SDS-PAGE

The recombinantly expressed proteins (*i.e*. FHL-1, CCP67 and CCP67± tail proteins) were assessed by SDS-PAGE to confirm they were of the expected size, purity and concentration (see Supplemental Figures S1 and S3). Two micrograms of protein were loaded and samples were run on 4–12% BisTris NuPAGE gels (ThermoScientific, Loughborough, UK) under reducing conditions. Blue Protein Standard Broad Range (New England Biolabs, Hitchin, UK) was used as a protein marker. Gels were stained using Coomassie Instant Blue (Expedeon, Swavesey, UK) for 1 hour at room temperature before being destained in miliQ water and photographed.

### Microtiter plate assays

#### CRP binding assays

CRP binding assays were adapted from published methods^20^. CRP (Sigma) was immobilized on Nunc-Immuno MaxiSorp F96-well plates (ThermoScientific) at 0.5 µg/well in Coating-Binding-Blocking (CBB) buffer (50 mM HEPES, 100 mM NaCl, pH 7.4) and coated overnight at 4 °C. Blank wells were coated with CBB buffer without protein. Since CRP dissociates from its pentameric form into monomers in the absence of calcium^64,65^, we immobilized CRP in both presence and absence of calcium to generate either pCRP or mCRP; moreover, for the experiments with pCRP, we included 2 mM CaCl_2_ in our CBB buffer. Between all steps plates were washed and dried three times in washing buffer (CBB buffer with 0.1% (v/v) Tween-20, pH 7.5). Following coating, plates were then blocked in 5% (w/v) BSA (Sigma) in CBB buffer for 1 hour at room temperature. FH/FHL-1 proteins were added in fluid phase at varying concentrations in CBB buffer for 2 hours at room temperature and bound protein was detected with the primary antibodies described above. Subsequently, either goat anti-mouse (against OX23) or goat anti-rabbit (against anti-CCP7) IgG coupled to alkaline phosphatase (Sigma) were added and incubated for 30 minutes. All primary and secondary antibodies/antisera, were added at a 1:2000 dilution in CBB buffer. Finally, 1 mg/ml p-nitrophenyl phosphate SigmaFast™ substrate (Sigma) was added to all wells and signals were measured at 405 nm (after appropriate development times) on a SpectraMax 340PC384 Microplate Reader (Molecular Devices, Sunnyvale, United States). Blank well values were subtracted from those from sample wells, where mean values from an average of two or three separate experiments were determined ± SEM.

Competition assays were also performed to determine if FHL-1 is able to compete for the binding of FH to mCRP. Here the assays were performed as described above but where the FH was kept constant (30 mM) being incubated with varying concentrations of FHL-1 (*i.e*. 4.7, 9.4, 18.8, 37.5, 75, 150, and 300 mM) for 30 min at room temperature before application to immobilized mCRP. Bound FH was subsequently detected with a goat anti-mouse monoclonal antibody against the C-terminal CCP20 domain of FH (clone L20/3, Cambridge Biosciences, Cambridge, UK) used at a dilution of 1:1000. Similarly, in competition assays where PTX3 competes for FHL-1 binding to mCRP, FHL-1 was kept constant at 50 mM and increasing PTX-3 concentrations (*i.e*. 7.8, 15.6, 31.25, 62.5, 128, 250, and 500 mM) were used. Bound FHL-1 was detected with the OX23 antibody as described above.

### PTX3 binding assays

PTX3 binding assays were carried out according to established protocols; in contrast to CRP assays, PTX3 was added in the solution phase. Varying concentrations of full-length FH, FHL-1, CCP6-8 and CCP67 ± tail proteins were coated on plates in PBS^+/+^ (PBS containing 0.90 mM CaCl_2_ and 0.49 mM MgCl_2_) overnight at 4 °C. Blank wells were coated with PBS^+/+^ without protein. Plates were blocked in 1% (w/v) BSA (Sigma) in wash buffer (20 mM TrisHCl, 150 mM NaCl, 1 mM MgCl_2_, 1 mM CaCl_2_, 0.1% (v/v) Tween-20, pH 7.0) for 2 hours at 37 °C. PTX3 was added in solution phase at 0.1 µg/well in wash buffer for 1 hour at 37 °C. Anti-PTX3 polyclonal antibody (diluted 1:10,000 in wash buffer) and subsequently goat anti-rabbit IgG coupled to alkaline phosphatase (Sigma-Aldrich; 1:2,000 dilution in wash buffer) was used to detect bound PTX3. Antibodies were incubated for 60 minutes at 37 °C. The plates were developed and read as described above. Between all steps plates were washed and dried three times in washing buffer.

### Donor Eye Tissue

Post-mortem donor eyes were obtained from the Manchester Eye Bank at the Royal Eye Hospital (Manchester, UK), within 48 hours from the time of death, after removal of the corneas for transplantation; these were classified and curated as part of the Manchester Eye Tissue Repository (ETR). No organs/tissues were procured from prisoners. Our research adhered to the tenets of the Declaration of Helsinki and in all cases, there was prior informed consent for the eye tissue to be used for research obtained and held by the Manchester Eye Bank, and guidelines established in the Human Tissue Act of 2004 (UK) were followed. Ethical approval for the use of tissue in these experiments was granted by the Manchester Eye Tissue Repository ethics committee (ref.^15^/NW/0932). Except in the case of AMD tissues (see below), none of the other donors had a history of visual impairment or eye disease; see Supplementary Table 1. Post mortem times for non-AMD and AMD donor tissue were similar with an average of 40 hours (range 28–48 h) and an average 37 hours (range 26–48 h) respectively. Six millimeter diameter punch biopsies were taken from the macula region of the retinas, and then fixed in 4% (v/v) paraformaldehyde for 1 hour, before being stored in OCT embedding matrix (Cell Path Ltd, Wales, UK) at −80 °C. All donor eyes were classified as AMD or non-AMD based on the presence of clinically sized drusen in the macula in post-mortem fundus images, which is carried out routinely within the Manchester ETR.

### Sample genotyping

#### DNA isolation

DNA was isolated from iris/ciliary body tissue dissected from post-mortem human donor eyes, using the Qiagen BioRobot® EZ1 and the EZ1 Advanced XL DNA Tissue Card (Qiagen, Crawley, UK). All samples were normalized to a concentration of 20 ng/μl for Sequenom analysis.

#### Assay design/SNP selection

A Sequenom assay was designed against potential SNPs implicated in AMD (*i.e*. *CFH* Y402H: rs1061170; *CFHR1-3* deletion: rs7542235; *ARMS2/HTRA1*: rs10490924). Primers were designed using Sequenom SNP Assay Design software version 3.0 for iPLEX reactions. The protocol and reaction conditions were in accordance with the manufacturer.

#### Genotyping and Sanger sequencing

Genotype identification was performed with the MassARRAY system from Sequenom. The MALDI-TOF MS spectra from the Sequenom MassARRAY^®^ Analyzer 4 instrument were analyzed using Typer 4.0.20 software (Sequenom, San Diego, USA). Automated fluorescent cycle DNA sequencing^66^ was performed using an Applied Biosystems model 3730 DNA analyzer. STADEN^67^ package Pregap4 1.4b1 software Version 1.8b1 (Medical Research Council, Laboratory of Molecular Biology, UK) were used for analysis of sequencing.

### Tissue sectioning

Macula tissues were sectioned with a Leica CM1950 cryostat (Leica Biosystems, Peterborough, UK) as described previously^10^. Briefly, sections were mounted with OCT embedding matrix (Cell Path Ltd, Wales, UK) onto pre-cooled chucks in the cryostat. The cryostat was kept at −18 °C, and 10 µm thick sections were made with a microtome blade (MX35 Premier Plus 34°/80 mm, Thermo Fisher Scientific, Waltham, MA, USA). Sections were mounted onto slides (X-tra Adhesive Clipped Corner Slides, Leica Biosystems, Peterborough, UK) and stored at −80 °C.

### Fluorescence immunohistochemistry

Tissue sections were prepared from the macular region as described above; see list of human donors in Supplementary Table 1. For CRP staining, five early AMD donors (average age = 74) were compared to six non-AMD donors (average age = 74). For PTX3 staining, eighteen early AMD donors (average age = 78) were compared to eighteen age-matched non-AMD donors (average age = 77). Immunofluorescent staining of eye sections was performed as previously published^7,10^. Briefly, slides were first incubated with chilled 50% (v/v) acetone, 50% (v/v) methanol (−20 °C) for 20 seconds. Slides were then washed thoroughly with PBS: all subsequent wash steps were performed at room temperature with PBS. Slides were then blocked by incubation with blocking solution (1 mg/ml BSA (Sigma), 1% (v/v) goat serum (Sigma), 0.1% (v/v) Triton X-100 (Sigma) in PBS) for 1 hour at room temperature. After thorough washing, primary antibodies against CRP (1:1000 in PBS) and PTX3 (1:10,000 in PBS) were added overnight at 4 °C. Slides were washed as above before the addition of the AlexaFlour488-conjugated secondary antibody for 2 hours at room temperature at a final concentration of 2 µg/ml in PBS. Sections were included where only secondary antibody was added as ‘blank’ controls. After a final wash with PBS, DAPI (Sigma-Aldrich; 1 µg/ml in PBS) was added and incubated for 5 min. Vectorshield (Vector Labs, Peterborough, UK) was added to preserve stained tissues before adding a coverslip. Slides were stored in the dark at 4 °C.

#### Equipment and settings

All images were collected on an *Olympus BX51* upright microscope using a 20x objective and captured using a Coolsnap ES camera (Photometrics) through MetaVue Software (Molecular Devices). Specific band pass filter sets for DAPI and FITC were used to prevent bleed through from one channel to the next. All LUTs are linear and cover the full range of data. Images were acquired at 8-bit resolution and analyzed using ImageJ64 (version 1.40 g; rsb.info.nih.gov/ij).

#### Fluorescence intensity measurements

For the quantitation of PTX3 staining in AMD and non-AMD donor eye tissue, previously described protocols were followed^10^. Briefly, gray scale images for both blank and stained sections for each donor were used for the measurement of relative fluorescent levels. Either the whole macula tissue (minus any RPE cells present) or just the choriocapillaris/Bruch’s membrane was selected using the selection tools in imageJ64. Between two and four separate fields of view per section were measured and averaged to determine a representative value for each donor. The final value obtained for the blank sections was subtracted from the value obtained for the respective test section to correct for the influence of donor-to-donor variance in tissue auto-fluorescence. Data were plotted and analyzed using GraphPad Prism (Version 6.04, GraphPad Software Inc.).

### Statistical analysis

Student’s T-tests were performed on solid phase binding assay data and a p-value <0.05 was considered significant; in addition, two-way ANOVA analysis was performed to analyze the binding of PTX3 to immobilized 402Y or 402 H variants of FHL-1. One-way ANOVA analysis was performed on PTX3 fluorescent staining intensities, where a p-value < 0.05 was considered significant.

## Electronic supplementary material

Supplementary informtaion
