# Supplementary material for: C-reactive protein and pentraxin-3 binding of factor H-like protein 1 differs from complement factor H: implications for retinal inflammation
Source: Sci Rep. 2018 Jan 26;8:1643. doi: 10.1038/s41598-017-18395-7 (PMC5786067; doi:10.1038/s41598-017-18395-7)
Supplement: Supplementary file 1 — Supplementary informtaion [file 41598_2017_18395_MOESM1_ESM.pdf]

C-reactive protein and pentraxin-3 binding of factor H-like protein 1 differs from complement factor H: implications for retinal inflammation

Maurice Swinkels, Justine H. Zhang, Viranga Tilakaratna, Graeme Black, Rahat Perveen, Selina McHarg, Antonio Inforzato, Anthony J. Day, Simon J. Clark

Supplementary Information

Supplementary Table 1: Donors used for fluorescent immunohistochemistry.

ETR number	Age	Sex	Post-mortem Time (hrs)	AMD status
ETR16	78	Female	32	AMD
ETR17	82	Male	45	Non-AMD
ETR19	79	Male	45	Non-AMD
ETR26	82	Female	37	AMD
**ETR37**	**75**	**Male**	**47**	**Non-AMD**
ETR50	74	Male	43	AMD
ETR51	78	Female	39	Non-AMD
**ETR62**	**76**	**Male**	**34**	**AMD**
ETR67	74	Male	32	Non-AMD
ETR68	80	Male	37	AMD
**ETR76**	**77**	**Female**	**44**	**Non-AMD**
ETR78	81	Female	39	AMD
ETR81	81	Female	48	Non-AMD
ETR84	84	Female	36	AMD
ETR86	78	Female	48	AMD
**ETR91**	**74**	**Male**	**42**	**AMD**
ETR92	82	Female	28	Non-AMD
**ETR94**	**77**	**Female**	**40**	**Non-AMD**
**ETR95**	**67**	**Female**	**28**	**Non-AMD**
**ETR99**	**67**	**Female**	**30**	**AMD**
ETR105	83	Female	26	AMD
**ETR107**	**81**	**Female**	**36**	**AMD**
ETR139	82	Male	31	AMD
ETR149	83	Female	30	Non-AMD
ETR151	72	Male	35	AMD
ETR152	72	Male	36	AMD
**ETR160**	**73**	**Male**	**30**	**AMD**
ETR170	70	Male	45	Non-AMD
**ETR180**	**74**	**Male**	**31**	**Non-AMD**
ETR187	77	Female	39	AMD
ETR188	74	Male	46	Non-AMD
ETR199	79	Female	43	Non-AMD
ETR200	79	Female	42	Non-AMD
ETR208	89	Female	46	AMD
ETR214	82	Female	48	Non-AMD
**ETR257**	**76**	**Male**	**38**	**Non-AMD**

Listed are the Manchester Eye Tissue Repository (ETR) numbers of the donors used for fluorescent immunohistochemistry, and their age, sex, AMD status and time post-mortem when macula tissues were processed. Macula from all donors of this list were stained for PTX3 (18 non-AMD, average age = 77; 18 early AMD, average age = 78), while only the donors in **bold** were stained for CRP (6 non-AMD, average age = 74; 5 early AMD, average age = 74).

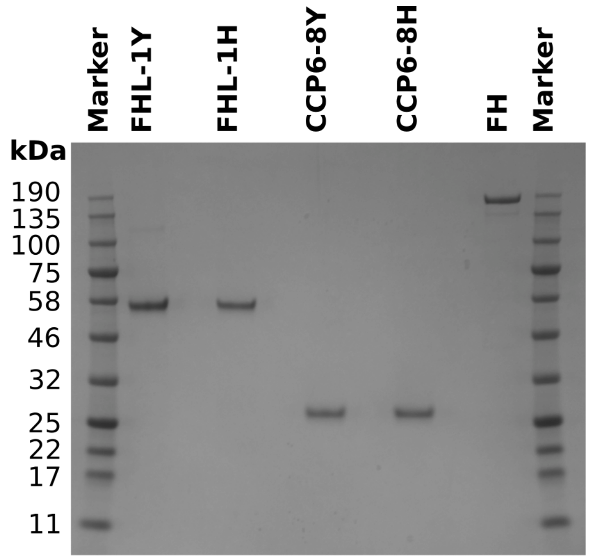

Figure S1. SDS-PAGE of FHL-1 and CCP6-8 recombinant proteins used in this study. Two micrograms each of in-house expressed recombinant FHL-1 and CCP6-8 (402H and 402Y variants in both cases) and commercially available FH were run on a 4-12% SDS-PAGE gel under reducing conditions. Proteins were stained with Instant Blue. This analysis indicates that the protein preparations used in the study are homogenous (single band per lane) and have the expected molecular weight (based on the apparent electrophoretic mobility); moreover, the 402H and 402Y protein pairs (for CCP6-8 and FHL-1) have equivalent concentrations, as also indicated by their absorbance at 280nm. Molecular weights of the protein markers are reported on the left.

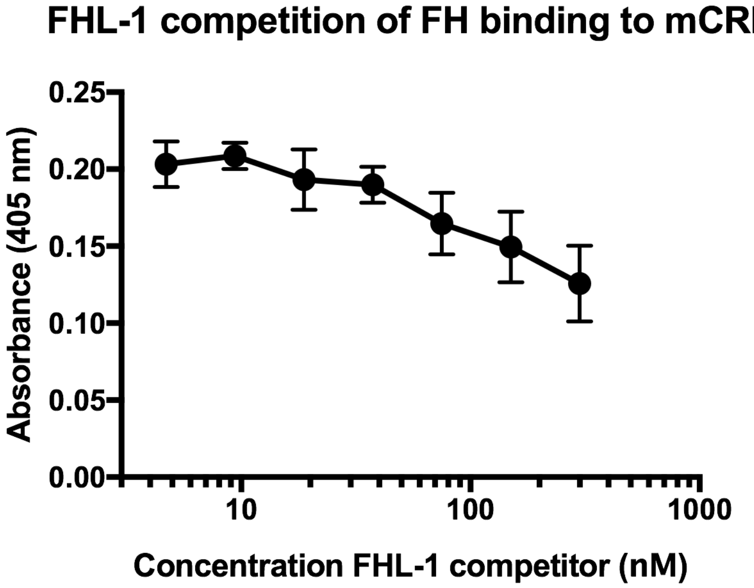

Figure S2. Competition of FH binding to mCRP by FHL-1. Increasing concentrations of FHL-1 (at 4.7, 9.4, 18.8, 37.5, 75, 150, and 300 mM) were combined with a constant amount of FH (30 mM) and added to immobilized mCRP. Bound FH was detected using an antibody against the C-terminal CCP20 domain (clone L20/3). Data (mean ± SEM) are from two independent experiments performed in quadruplicate (n = 8).

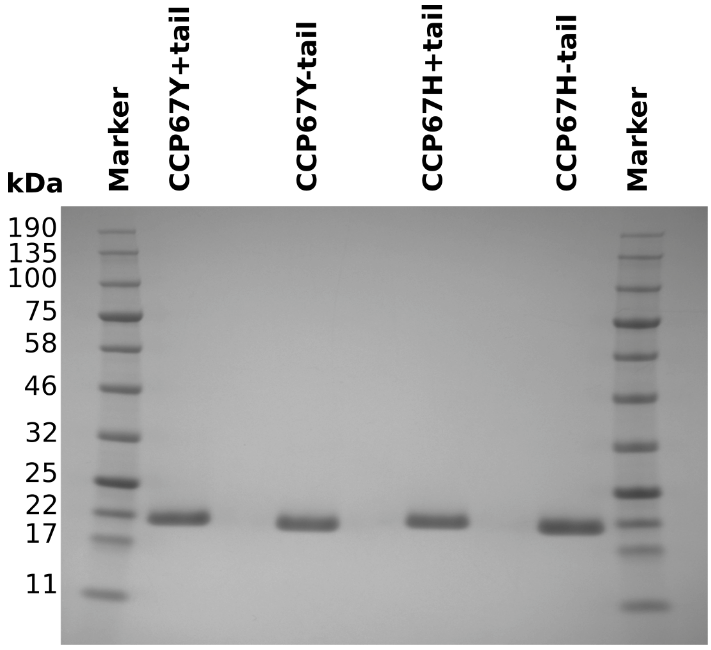

Figure S3. SDS-PAGE of CCP67±tail recombinant proteins. Two micrograms each of in-house expressed CCP67 proteins (both 402Y and 402H variants plus and minus C-terminal tail) were run on a 4-12% SDS-PAGE gel under reducing conditions, to verify their purity and relative concentrations. Proteins were stained with Instant Blue as in Figure S1. Molecular weights of the protein markers are shown on the left.

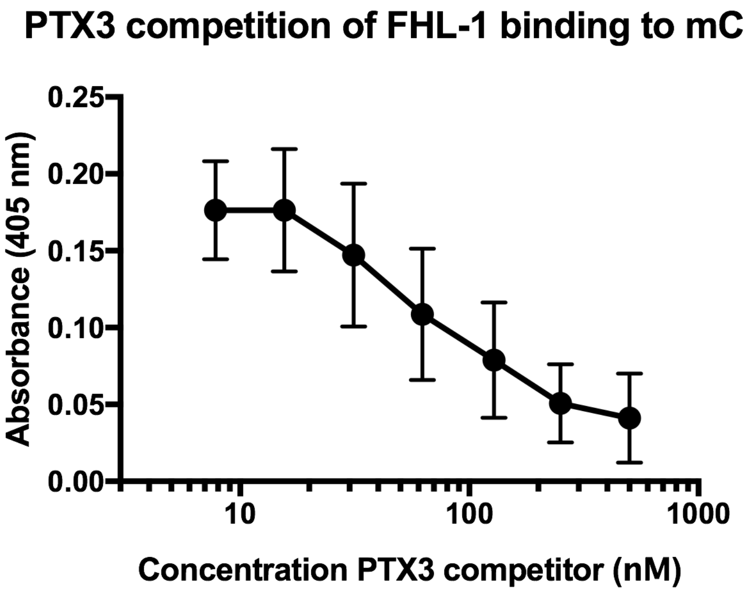

Figure S4. Competition of FHL-1 binding to mCRP by PTX3. Increasing concentrations of PTX3 (at 7.8, 15.6, 31.3, 62.5, 128, 250, and 500 mM) were combined with a constant concentration of FHL-1 (50 mM) and added to immobilized mCRP. Bound FHL-1 was detected using the OX23 monoclonal antibody. Data (mean ± SEM) are from two independent experiments performed in quadruplicate (n = 8).

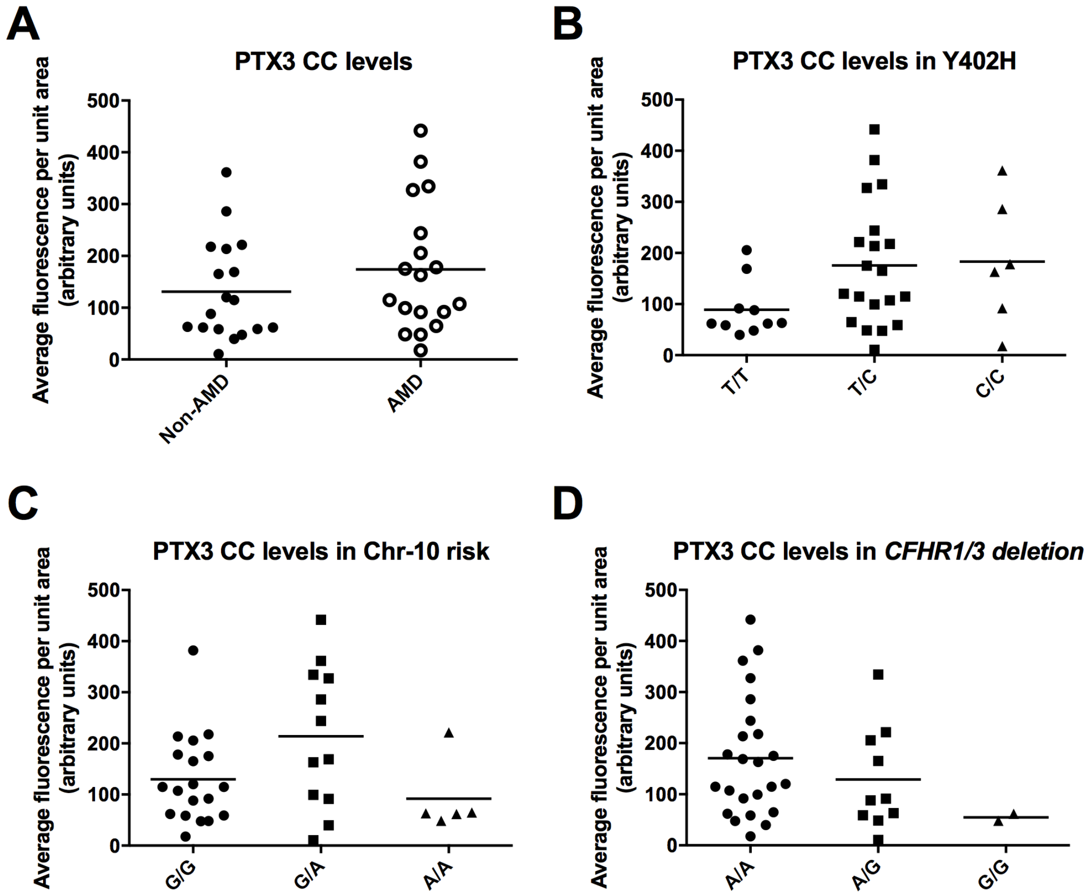

Figure S5. Analysis of PTX3 staining in the choriocapillaris of human donor eyes stratified for AMD and AMD-risk genotypes. The average fluorescence intensity of PTX3 staining in the choriocapillaris (CC) of eighteen non-AMD donors and eighteen AMD donors is shown; these are the same donors as analyzed in Figure 4. **A**, The levels of PTX3 staining in the choriocapillaris of AMD eyes compared to age-matched control eyes. **B**, Comparison of PTX3 staining levels in the choriocapillaris of donor eyes stratified based on Y402H risk genotype; T/T is the non-risk genotype (corresponding to 402Y/402Y), and C/T and C/C (corresponding to 402H/402H) are AMD genotypes of increasing risk. **C**, PTX3 staining levels stratified based on risk genotype at chromosome 10 (Chr-10), where G/G is the non-risk genotype and A/G and A/A are AMD-associated. **D**, PTX3 levels in the choriocapillaris stratified for the protective *CFHR1/3* gene deletion (G-allele). Statistical analysis was performed using one-way ANOVA.
